# Increased epigenetic age and granulocyte counts in the blood of Parkinson's disease patients

**DOI:** 10.18632/aging.100859

**Published:** 2015-12-09

**Authors:** Steve Horvath, Beate R. Ritz

**Affiliations:** ^1^ Department of Human Genetics, David Geffen School of Medicine, University of California Los Angeles, Los Angeles, CA 90095, USA; ^2^ Department of Biostatistics, UCLA Fielding School of Public Health, University of California Los Angeles, Los Angeles, CA 90095, USA; ^3^ Department of Neurology, UCLA School of Medicine, University of California Los Angeles, Los Angeles, CA 90095, USA; ^4^ Department of Epidemiology, UCLA Fielding School of Public Health, University of California Los Angeles, Los Angeles, CA 90095, USA; ^5^ Department of Environmental Health, UCLA Fielding School of Public Health, University of California Los Angeles, Los Angeles, CA 90095, USA

**Keywords:** Parkinson's disease, epigenetics, epigenetic clock, DNA methylation, granulocyte, neutrophil

## Abstract

It has been a long standing hypothesis that blood tissue of PD Parkinson's disease (PD) patients may exhibit signs of accelerated aging. Here we use DNA methylation based biomarkers of aging (“epigenetic clock”) to assess the aging rate of blood in two ethnically distinct case-control data sets. Using n=508 Caucasian and n=84 Hispanic blood samples, we assess a) the intrinsic epigenetic age acceleration of blood (IEAA), which is independent of blood cell counts, and b) the extrinsic epigenetic age acceleration rate of blood (EEAA) which is associated with age dependent changes in blood cell counts. Blood of PD subjects exhibits increased age acceleration according to both IEAA (p=0.019) and EEAA (p=6.1×10^−3^). We find striking differences in imputed blood cell counts between PD cases and controls. Compared to control subjects, PD subjects contains more granulocytes (p=1.0×10^−9^ in Caucasians, p=0.00066 in Hispanics) but fewer T helper cells (p=1.4×10^−6^ in Caucasians, p=0.0024 in Hispanics) and fewer B cells (p=1.6×10^−5^ in Caucasians, p=4.5×10^−5^ in Hispanics). Overall, this study shows that the epigenetic age of the immune system is significantly increased in PD patients and that granulocytes play a significant role.

## INTRODUCTION

The progressive motor and non-motor decline in Parkinson's disease (PD) leads to disability and loss of quality of life. Onset is insidious with some non-motor symptoms occurring years before diagnosis [1]. The extensive loss of dopamine neurons prior to diagnosis makes early interventions the ultimate treatment goal. For this to become reality, development of biomarkers with the potential of early detection and treatment of PD during a period coined the ‘molecular prodrome’ is critical [2]. While blood provides an easily accessible tissue, biomarker development based on it has remained an elusive goal. Even though substantial evidence exists that inflammation contributes to the pathogenesis of PD [3], the question remains whether blood tissue also reflects earliest changes in PD such that blood cell counts or immune markers can help predict onset or even become a treatment target. This idea inspired research as early as 1985, when Marttila et al. observed that PD patients exhibit signs of immune suppression partially resembling those seen in normal aging but being quantitatively exaggerated with a decrease in helper (CD4+) T cells [4].

Until recently relatively few molecular biomarkers of aging have lent themselves for rigorously testing whether PD is associated with accelerated aging in the immune system and blood. Telomere length can be used as a molecular aging marker but its association with PD status remains ambiguous despite considerable research effort. A keyword search for [“telomere” AND “Parkinson's disease”] in Pubmed led to the identification of 10 articles. After eliminating a commentary and a study of mice, we reviewed references [5–12]. Two of these studies did not find an association between telomere length and PD status [5, 8]. Three studies, including the largest study to date [12], found borderline significant (P=0.02) associations in the opposite direction from what would be expected, i.e. counter to the aging hypothesis PD cases had longer telomeres [6, 9, 12]. Here, we answer the challenge to explore these counterintuitive results observed in a methodologically strong study by exploiting an entirely new class of molecular biomarker of aging based on epigenetic data. Several recent studies have proposed to measure the physiological age of tissue samples by combining the DNA methylation levels of multiple dinucleotide markers, known as Cytosine phosphate Guanines or CpGs [13–15]. In particular, the epigenetic clock (based on 353 CpG markers) was developed to measure the age (known as “DNA methylation age” or “epigenetic age”) of sorted human cell types (CD4+T cells or neurons), tissues, and organs—including blood, brain, breast, kidney, liver, lung [14], and even prenatal brain samples [16].

The epigenetic clock method -applied to two commercially standardized methylation platforms: the Illumina 450K array and the 27K arrays - is an attractive biomarker of aging because (1) it applies to most human tissues; (2) its accurate measurement of chronological age is unprecedented [14]; (3) it is predictive of all-cause mortality even after adjusting for a variety of known risk factors [17]; (4) it correlates with measures of cognitive and physical fitness in the elderly [18]; and (5) it has already been useful in detecting accelerated aging due to obesity [19], Down syndrome [20], and HIV infection [21]. Further, the epigenetic clock was used to show that age acceleration of blood may predict the future onset of lung cancer [22], the cerebellum ages slowly [23], and 3) that the blood of subjects with a severe developmental disorder ages normally [24]. Despite many diverse applications of the epigenetic clock [16, 25–27], we are not aware of any study that related epigenetic age acceleration to PD status.

In this large epigenetic study of PD, we show for the first time that measures of epigenetic age acceleration are associated with PD status. Different from typical epigenome wide association studies (EWAS) that interrogate individual CpGs, the current study posits two broad hypotheses: First, that a measure of epigenetic age acceleration is associated with PD status. Second, that (imputed) measures of blood cell types (based on DNA methylation levels) are associated with PD status. To address these hypotheses, we leverage a large and unique community-based case control study described in the following.

## RESULTS

### Study design and study population

The Parkinson's disease, Environment, and Genes (PEG) case-control study aims to identify environmental risk factors (e.g. neurotoxic pesticide exposures) for Parkinson's disease. The PEG study is a large population-based study of Parkinson's disease of mostly rural and township residents of California's central valley [28]. Cases were identified with the help of local neurologists, clinics, and community outreach and controls were randomly sampled from Medicare lists and residential tax assessor's records. All covariates were ascertained in interviews with subjects.

Every PD patient was evaluated by a UCLA movement disorder specialist. Most subjects were seen multiple times. Blood was drawn early in the disease, on average 1.5 years after PD diagnosis. We only used DNA samples from wave 1 (PEG1).

In our analysis we started out with analyzing all subjects (irrespective of race/ethnicity). Next we focused on specific ethnic strata (Caucasians only or Hispanics only).

### DNA methylation data sets

The first blood DNA methylation data set was comprised of 508 Caucasians (non-Hispanic whites) and the second of 84 Hispanics enrolled in the PEG study, respectively. Descriptive information for the data sets we used can be found in Table 1. When we related various demographic and known risk factors to PD status in a marginal analysis, education was associated positively (p=0.0085 in Caucasians, p=0.27 in Hispanics, Table 1) and smoking negatively with PD in this subsample. The first association reflects a well-known ascertainment bias in epidemiological case-control studies: healthy controls with little incentive to participate in research tend to be more highly educated. For smoking, our finding is consistent with the literature which is reviewed and discussed in [29]. Our marginal analysis also shows that exposure to pesticides (total organophosphate count in residential or occupational settings) is strongly associated with an increased PD risk (p=4×10–^6^ Table 1) consistent with our previous publications [30].

**Table 1:** Overview of the two DNA methylation data sets

	Caucasian (Data Set 1)			Hispanic (Data Set 2)		
	P-value	PD	control	P-value	PD	control
**Sample size**		289	219		46	38
**No. Female**	0.47	125	102	0.05	14	20
**Smoking Status**	0.013			0.21		
Smoking: current		15	13		3	4
Smoking: former		120	118		21	6
Smoking: never		154	88		22	8
	**P-value**	**mean (SE), min, max**	**mean (SE), min, max**	**P-value**	**mean (SE), min, max**	**mean (SE), min, max**
**Smoking: total pack years**	0.0051	11.4 (1.3),0,175	14.8 (1.6),0,125	0.65	5.7 (2),0,73	7 (2.9),0,39
**Age**	0.053	71 (0.6),37,91	68 (0.8),35,92	0.60	67.3 (1.6),37,83	65 (2.1),36,86
**Year Born**	0.14	1932 (0.6),1915,1966	1935 (0.8),1912,1969	0.27	1938 (2),1920,1964	1944 (4),1918,1969
**Education: no. of years in school**	0.0085	14.1 (0.2),6,30	14.8 (0.2),5,27	0.16	9.6 (0.72),0,20	11.2 (1),1,19
**PD Family History**	0.16	0.15 (0.021),0,1	0.11 (0.021),0,1	0.30	0.15 (0.05),0,1	0.6 (0.056),0,1
**Caffeinated Coffee: lifetime weighted ave. (cup/day)**	0.21	1.8 (0.12),0,14	2.2 (0.18),0,19	0.54	1.6 (0.48),0,20	1.3 (0.29),0,4
**Organophospate count (residential+occuputation)**	2.8×10–8	9 (0.59),0,46	4.9 (0.47),0,41	1.7×10–3	13.3 (1.63),0,37	5.9 (2.4),0,30
**Year diagnosed with PD**		2001 (0.3),1998,2007			2002 (0.3),1998,2006	
**Levodopa Medication status**		0.7 (0.03)			0.67 (0.v07)	
**Levodopa mg/day**		350 (16),0,2300			369 (41),0,1020	

Data sets 1 and 2 are comprised of Caucasians (non-Hispanic whites) and Hispanics, respectively. The p-value resulted from relating the respective variables to PD status using a non-parametric group comparison test (Kruskal Wallis test) or Fisher's exact test (for categorical variables).

### Accuracy of the epigenetic clock

DNAm age (also referred to as epigenetic age) was calculated as described in [14] from human samples profiled with the Illumina Infinium 450K platform. The epigenetic clock is defined as a prediction method of age based on the DNAm levels of 353 CpGs. Predicted age, referred to as DNAm age, correlates with chronological age in sorted cell types (CD4 T cells, monocytes, B cells, glial cells, neurons), tissues and organs, including: whole blood, brain, breast, kidney, liver, lung, saliva [14]. Mathematical details and software tutorials for the epigenetic clock can be found in the Additional files of [14]. An online age calculator can be found at our webpage (https://dnamage.genetics.ucla.edu). All of the described epigenetic measures of aging and age acceleration are implemented in our freely available software.

**Figure 1:**
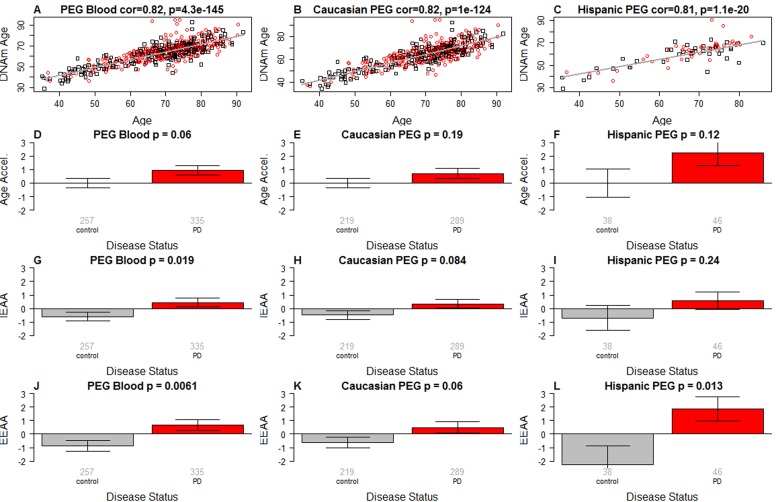
Epigenetic age analysis of PD (**A**-**C**) DNA methylation age (y-axis) versus chronological age (x-axis) in (A) all subjects, (**B**) Caucasians only, and (**C**) Hispanics only. Dots corresponds to subjects and are colored by PD disease status (red=PD, black=control). We define three measures of epigenetic age acceleration. (**D**-**F**) presents results for the “universal” measure of epigenetic age acceleration, which is defined as residual to a regression line through the control samples, i.e. the vertical distance of a point from the line. By definition, the mean age acceleration in controls is zero. (**G**-**I**) The bar plots relate measures of intrinsic epigenetic age acceleration to PD status. This measure is independent of blood cell counts. The fourth row (panels **J**-L) reports findings for the measure of extrinsic epigenetic age acceleration, which does relate to changes in cell composition. Each bar plot depicts the mean value (y-axis), 1 standard error, and the group size (underneath the bar). The p-value results from a Student T-test.

As expected, DNAm age has a strong linear relationship with chronological age (Figure 1). The high accuracy of the epigenetic clock is validated in both data sets in which DNAm age is highly correlated with chronological age (r=0.82 in Caucasians; r=0.81 in Hispanics, Figure 1A-C).

### Three measures of epigenetic age acceleration

In this article, we consider three measures of epigenetic age acceleration (as detailed in Methods). The first measure, which will be referred to as universal measure of age acceleration (denoted *AgeAccel*) applies to virtually all tissues and cell types (with the exception of sperm) [25]. The other two measures (referred to as intrinsic and extrinsic age acceleration, respectively) only apply to blood. The universal measure *AgeAccel* is defined as the difference between DNAm age value and the value predicted by a spline regression model in controls.

The measure of intrinsic epigenetic age acceleration (IEAA) measures “pure” epigenetic ageing effects in blood that are not confounded by differences in blood cell counts. The measure of *extrinsic* epigenetic age acceleration (EEAA) aims to measure ageing in immune related components also relates to age related changes in blood cell composition such as the decrease of naive CD8+ T cells and the increase in memory or exhausted CD8+ T cells [31–33]. EEAA is defined on the basis of a weighted average of the epigenetic age measure from Hannum et al (2013) [13] and three blood cell types that are known to change with age: naive (CD45RA+CCR7+) cytotoxic T cells, exhausted (CD28-CD45RA-) cytotoxic T cells, and plasma B cells. By definition, EEAA has a positive correlation with the amount of exhausted CD8 T cells and plasma blast cells and a negative correlation with the amount of naive CD8+ T cells. Blood cell counts were estimated based on DNA methylation data as described in the section entitled “Estimating blood cell counts based on DNA methylation levels”. The three different measures of epigenetic age acceleration are not independent of each other. The universal measure AgeAccel is correlated with IEAA (r=0.90 in Caucasians and r=0.77 in Hispanics) and with EEAA (r=0.55 in Caucasians and r=0.74 in Hispanics). IEAA is also correlated with EEAA (r=0.41 in Caucasians and again r=0.41 in Hispanics). By construction, our three measures of epigenetic age acceleration are uncorrelated (r=0) with chronological age at the time of blood draw.

### PD is associated with intrinsic and extrinsic epigenetic age acceleration

PD status has a (marginally) significant relationship with all 3 measures of age acceleration: p=0.06 for the universal measure of age acceleration (Figure 1A-C), p=0.019 for IEAA (Figure 1G-I), and p=0.0061 for EEAA (Figure 1J-L).

**Figure 2:**
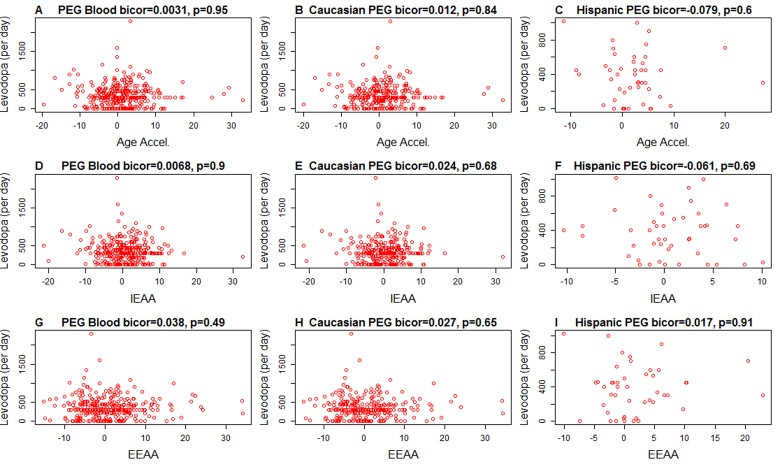
Levodopa medication (x-axis) versus epigenetic age acceleration in PD subjects Each scatter plot depicts the amount of levodopa medication (milligram per day) versus (**A,B,C**) universal epigenetic age acceleration, (**D,E,F**) intrinsic epigenetic age acceleration (**G,H,I**), extrinsic epigenetic age acceleration. The first, second, and third column correspond to all subjects, Caucasians only, and Hispanics only, respectively. Each dot (PD patient) is colored in red for the sake of consistency with Figure 1. The heading of each plot reports a robust correlation coefficient (biweight midcorrelation and a corresponding p-value).

**Figure 3:**
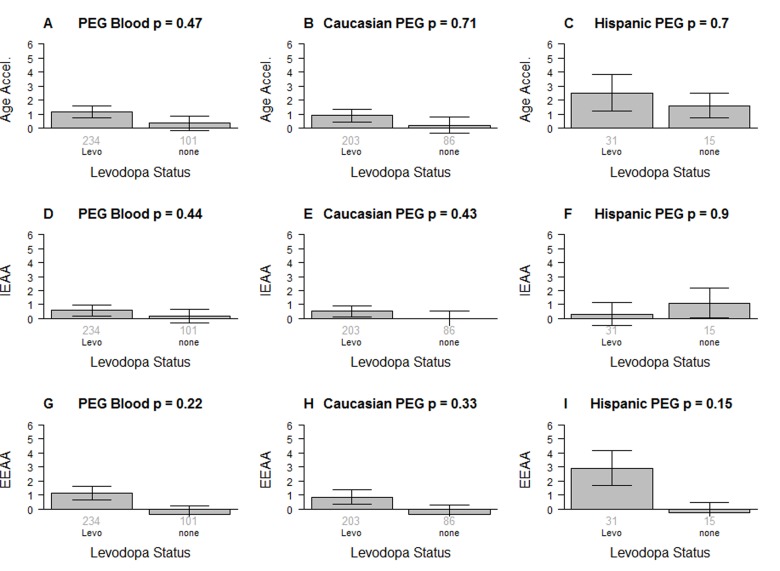
Levodopa medication status versus epigenetic age acceleration in PD patients The first, second, and third column correspond to all subjects, Caucasians only, and Hispanics only, respectively. Levodopa medication status versus (**A,B,C**) universal epigenetic age acceleration, (**D,E,F**) intrinsic epigenetic age acceleration (**G,H,I**), extrinsic epigenetic age acceleration. Each bar plot depicts the mean value (y-axis), 1 standard error, and the group size (underneath the bar). The p-value results from a non-parametric group comparison test (Kruskal Wallis).

It is unlikely that Levodopa medication explains the increased epigenetic age acceleration since we find no significant association between the amount of Levodopa medication and any of the measures of age acceleration in PD patients (Figure 2). These results were corroborated in a second analysis in which we related medication status (binary grouping variable) to the measures of epigenetic age acceleration in PD patients (Figure 3) and found no associations.

None of the measures of epigenetic age acceleration were significantly associated with smoking status, pesticide exposure, or family history of PD; however, sex had a significant association: compared to men, women have a low EEAA (p-value=2.9×10–^6^ in Caucasians and p=0.016 in Hispanics) and a low IEAA (p=0.0050 in Caucasians, p=0.35 in Hispanics). By study design, sex was not associated with PD status in Caucasians (p=0.45) but there was a marginally significant association in Hispanics (p=0.04). Family history of PD was not predictive of PD status.

In a multivariate logistic regression analysis with PD status as the outcome we find that AgeAccel (p=0.037) remains a significant covariate even after adjusting for chronological age (at the time of blood draw), blood cell counts, pesticide exposure (organophosphate), smoking (cumulative pack years), education (number of years in school), coffee consumption (life time measured as a weighted average cup per day), and ethnicity. In an analogous model, IEAA is only marginally significant (p=0.084, Table 2). EEAA is significantly associated with PD status (p=0.031, Table 2) after adjusting for chronological age, pesticide exposure (organophosphate), smoking (cumulative pack years), education (number of years in school), coffee consumption (life time measured as a weighted average cup per day), and ethnicity.

**Table 2:** Logistic model that regresses PD status on covariates

Logistic model. Outcome= PD	Measure=AgeAccel			Measure=IEAA			Measure=EEAA		
Covariates	Coef	SE	P-value	Coef	SE	P-value	Coef	SE	P-value
Age	0.016	0.0085	0.061	0.015	0.0085	0.071	0.023	0.008	0.004
Measure of Age Acceleration	0.036	0.017	0.037	0.031	0.018	0.084	0.031	0.014	0.031
Granulocyte	3.5	1.6	0.027	2.7	1.5	0.07			
CD4+T cell	−3.8	2.3	0.11	−4.9	2.3	0.029			
CD8+T cell	−1.6	3	0.59	−1.3	3	0.67			
Organo phosphate exposure	0.054	0.012	4E-6	0.055	0.012	3.7E-6	0.059	0.012	4E-7
Smoking (total pack years)	−0.0081	0.0043	0.063	−0.0082	0.0043	0.06	−0.0077	0.004	0.067
Number of years in school	−0.058	0.028	0.036	−0.058	0.028	0.035	−0.049	0.027	0.064
LifetimeCoffee (ave cup/day)	−0.035	0.041	0.4	−0.034	0.041	0.41	−0.037	0.04	0.36
Ethnicity(Hispanic)	0.32	0.35	0.36	0.31	0.35	0.37	0.3	0.34	0.37

To estimate the actual amount of age acceleration, we regressed DNAm age on disease status, age, granulocytes, smoking, ethnicity, and sex. According to this multivariate regression model, the blood of PD patients is 1.5 years older than that of age matched controls.

### PD patients have more granulocytes but fewer helper T cells and B cells than controls

**Figure 4:**
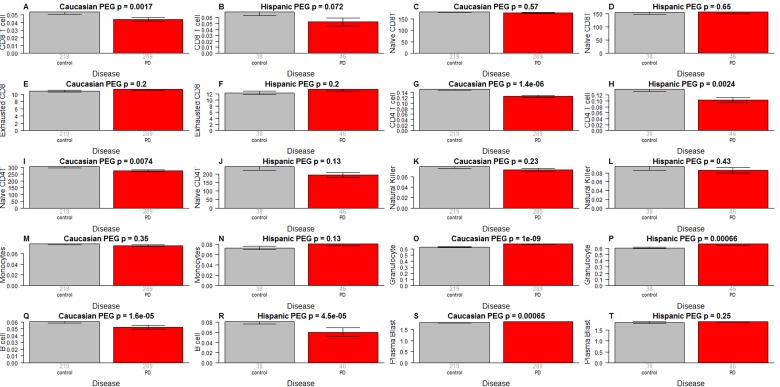
Blood cell counts versus PD status As indicated in the heading of each panel, the panels alternate between the two data sets. PD status (x-axis) versus (**A,B**) proportion of cytotoxic CD8+ T cells, (C,D) naïve CD8+ T cell count, (**E,F**) percentage of exhausted CD8+ T cells (defined as CD8+CD28-CD45RA-), (**G,H**) proportion of helper CD4+ T cells, (**I,J**) naïve CD4+ T cell count, (**K,L**) proportion of natural killer cells, (**M,N**) proportion of monocytes, (**O,P**) granulocytes, (**Q,R**) B cells, (**S,T**) plasma blasts (activated B cells). The abundance measures of blood cell counts were estimated based on DNA methylation levels using the epigenetic clock software. The y-axis of (**E,F**) reports a percentage, that of (**C,D,I,J**) a cell counts but it is best to interpret these measures as ordinal abundance measures. The y-axis of the other panels reports estimated proportions based on the Houseman method [45]. Each bar plot depicts the mean value (y-axis), 1 standard error, and the group size (underneath the bar). The p-value results from a non-parametric group comparison test (Kruskal Wallis).

We find striking differences in blood cell composition between PD cases and controls (Figure 4). Compared to control samples, PD patients have more granulocytes (p=1.0×10–^9^ in Caucasians, p=0.00066 in Hispanics Figure 4O,P) and plasma cells (activated B cells) (p=0.00065 in Caucasians Figure 4S) but fewer helper (CD4+) T cells (p=1.4×10–^6^ in Caucasians, p=0.0024 in Hispanics, Figure 4G,H), fewer naïve CD4+ T cells (p=0.0074 in Caucasians, p=0.13 in Hispanics Figure 4I,J), fewer B cells (p=1.6×10–^5^ in Caucasians, p=4.5×10–^5^ in Hispanics Figure 4Q,R), and fewer cytotoxic (CD8+) T cells (p=0.0017 in Caucasians, p=0.072 in Hispanics Figure 4A,B). A multivariate logistic regression analysis shows that granulocyte count remains a significant predictor of PD status (p=0.027, Table 2) even after adjusting for other covariates. We did not observe significant association between PD status and the amount of naïve CD8+ T cells (Figure 4C,D), exhausted CD8+ T cells (Figure 4E,F), natural killer cells (Figure 4K,L) or monocytes (Figure 4M,N).

**Figure 5:**
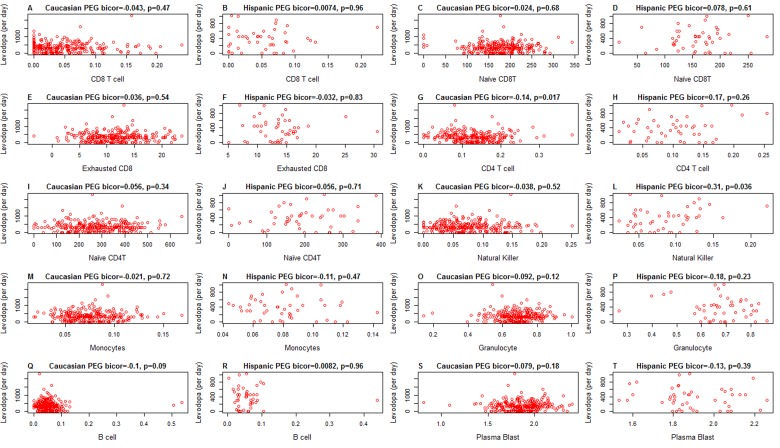
Amount of medication (x-axis) versus epigenetic age acceleration in PD subjects As indicated in the heading of each panel, the panels alternate between the two data sets. PD status (x-axis) versus (**A,B**) proportion of CD8+ T cells, (**C,D**) naïve CD8+ T cell count, (**E,F**) exhausted CD4+T cell counts (defined as CD8+CD28-CD45RA-), (**G,H**) proportion of CD4+ T cells, (**I,J**) naïve CD4 T cell count, (**K,L**) proportion of natural killer cells, (**M,N**) proportion of monocytes, (**O,P**) granulocytes, (**Q,R**) B cells, (**S,T**) plasma blasts (activated B cells). All cell types were estimated based on DNA methylation levels as described in Methods. The heading of each plot reports a robust correlation coefficient (biweight midcorrelation and a corresponding p-value).

**Figure 6:**
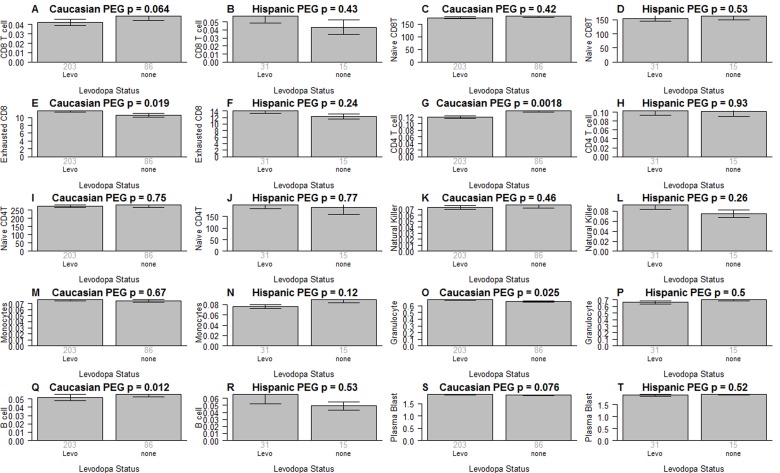
Medication status versus blood cell counts in PD patients As indicated in the heading of each panel, the panels alternate between the two data sets. Levodopa medication status (x-axis) versus (**A,B**) proportion of CD8+ T cells, (**C,D**) naïve CD8+ T cell count, (**E,F**) exhausted CD+T cell counts (defined as CD8+CD28-CD45RA-), (**G,H**) proportion of CD4+ T cells, (**I,J**) naïve CD4+ T cell count, (**K,L**) proportion of natural killer cells, (**M,N**) proportion of monocytes, (**O,P**) granulocytes, (**Q,R**)B cells, (**S,T**) plasma blasts (activated B cells). All cell types were estimated based on DNA methylation levels as described in Methods. Each bar plot depicts the mean value (y-axis), 1 standard error, and the group size (underneath the bar). The p-value results from a non-parametric group comparison test (Kruskal Wallis).

It is unlikely that medications explain the difference in blood cell counts because both medication status and amount of medication have no more than a weak association with blood cell counts in PD subjects (Figure 5): when relating the amount of Levodopa (mg per day) to blood cell counts in PD subjects, we only found a weak marginally significant correlation with CD4+ T cells (r=−0.14, p=0.017 in Caucasians, Figure 5G). We only observed relatively weak associations between Levodopa medication status (binary) and blood cell counts (Figure 6). In Caucasian PD patients, we found that medicated patients have fewer CD4+ T cells (p=0.0018 Figure 6G), granulocytes (p=0.025 Figure 6O), and B cells (p=0.012 Figure 6Q) but more exhausted CD8+ T cells (p=0.019 Figure 6E). In Hispanic PD patients, we could not detect a significant association between medication status and blood cell counts, which might reflect the small number (n=15) of un-medicated PD patients in this group.

## DISCUSSION

We and others have shown that epigenetic biomarkers of aging based on genome-wide DNA methylation levels are highly robust and reproducible (see also Figure 1A,B) [13–15, 17, 19]. We use these biomarkers of aging to explore the contributions of aging in a large community-based study of PD. Ours is the first data substantiating the longstanding hypothesis regarding accelerated aging effects in PD using epigenetic biomarkers of aging. PD status has a significant relationship with all 3 measures of age acceleration but the strongest associations can be observed for the extrinsic measure EEAA, which also keeps track of age related changes in blood cell composition.

However, the observed accelerated aging effects do not simply reflect changes in blood cell composition as can be seen from the fact that PD subjects also exhibit increased intrinsic epigenetic aging rates.

Our study demonstrates an unexpectedly strong association between granulocytes and PD status. Several previous studies evaluated blood cell counts in PD subjects using flow cytometric method [34–38]. The most recent study including the largest number of patients yet (88 PD cases and 77 age-gender matched controls)[38] reported reduced numbers of T helper and B lymphocytes in Parkinson's disease. Our study corroborates these findings for T helper (p=1.4×10^−6^) and B cells (p=1.6×10^−5^ Figure 4Q) however, granulocytes exhibited a far more significant association with PD status (p=1.0×10^−9^ Figure 4O) in our population. Our study does not allow us to identify the type of granulocytes (neutrophil, eosinophil, or basophil) with the strongest effect. Yet, given the abundance of neutrophils (~60% of all blood cells compared with 0.5–2.5% for eosinophils and basophils) we suspect that they are responsible for the signal we saw in blood. An increased neutrophil/lymphocyte ratio has been observed in PD subjects [39] and differential neutrophil infiltration has been shown to contribute to regional differences with brain inflammation favoring the substantia nigra pars compacta over the cortex [40]. We acknowledge the following limitations. First, the one-time only blood sampling protocol early in disease does not allow us to establish temporality of cause and effect. We hypothesize that accelerated aging of the immune system and/or altered blood cell counts (including neutrophils) precede the onset of motor and cognitive symptoms in PD but future studies are needed to determine whether these blood based biomarkers are prognostic of incident PD.

Second, we necessarily focused only on blood tissue. Future studies should evaluate whether accelerated epigenetic aging effects can also be found in other tissues (notably brain tissue).

Finally, we did not relate individual CpGs to PD status since this is beyond the scope of this article which focuses on epigenetic aging effects and blood cell counts. We refer the reader to future publications from our group and other DNA methylation studies of Parkinson disease (PD) and related disorders [41–43]. Our study also has strengths including a novel data set for two distinct ethnic populations, a large sample size (total n=592), powerful epigenetic biomarkers of aging, an unprecedented breadth of blood cell counts, a community (population)-based design, and extensive clinical evaluations by movement disorder specialists to establish PD diagnoses.

Increased levels of epigenetic age acceleration or blood cell counts are not specific to PD but our results may inform the future development of DNA methylation based biomarkers of PD. Overall, our results support the notion that neuroinflammation, which leads to brain cell death and PD disease progression, is fueled by activated glial cells communicating with peripheral immune cells.

## METHODS

### Ethics review and IRB

Informed consent was obtained from all subjects. This study was reviewed by the UCLA institutional review board (IRB#13–000671 and IRB#14–000061).

### Preprocessing of Illumina Infinium 450K arrays

In brief, bisulfite conversion using the Zymo EZ DNA Methylation Kit (ZymoResearch, Orange, CA, USA) as well as subsequent hybridization of the HumanMethylation450k Bead Chip (Illumina, SanDiego, CA), and scanning (iScan, Illumina) were performed according to the manufacturers protocols by applying standard settings. DNA methylation levels (β values) were determined by calculating the ratio of intensities between methylated (signal A) and unmethylated (signal B) sites. Thus, β values range from 0 (completely un-methylated) to 1 (completely methylated).

### Measures of epigenetic age acceleration

The name of our universal measure of age acceleration (*AgeAccel*) reflects that it applies to virtually all sources of human DNA (with the exception of sperm). Here we defined it as follows. First, we regressed DNAm age on chronological age in controls. Next, we used the resulting model to predict the DNAm age of each subject. Next the universal measure was defined as the difference between the observed measure of DNAm age and the predicted value. Thus, a high positive value for *AgeAccel* indicates that the observed DNAm age is higher than that predicted based on controls. *AgeAccel* has a relatively weak correlation with blood cell counts [21] but it still relates to blood cell counts. To subtract out the effect of blood cell counts, we find it useful to define a measure of intrinsic epigenetic age acceleration (IEAA) which measures “pure” epigenetic ageing effects that are not confounded by differences in blood cell counts. It is defined as the residual resulting from a multivariate regression model of DNAm age on chronological age and various blood immune cell counts (naive CD8+ T cells, exhausted CD8+ T cells, plasma B cells, CD4+ T cells, natural killer cells, monocytes, and granulocytes).

The measure of *extrinsic* epigenetic age acceleration (EEAA) aims to measure epigenetic ageing in immune related components. EEAA is defined using the following three steps. First, we calculated the epigenetic age measure from Hannum et al (2013) [13] based on 71 CpGs. The resulting age estimate is correlated with certain blood cell types [17]. Second, we increased the contribution of blood cell types to the age estimate by forming a weighted average of the Hannum estimate with 3 cell types that are known to change with age: naive (CD45RA+CCR7+) cytotoxic T cells, exhausted (CD28-CD45RA-) cytotoxic T cells, and plasma B cells using the approach of [44]. The resulting measure of blood age is referred to as BioAge4 in our epigenetic clock software. Third, we defined a measure of age acceleration (EEAA) as the residual resulting from a univariate model regressing BioAge4 on chronological age. By definition, our measure of EEAA has a positive correlation with the amount of exhausted CD8+ T cells and plasma blast cells and a negative correlation with the amount of naive CD8+ T cells. Blood cell counts were estimated based on DNA methylation data as described in the section entitled “Estimating blood cell counts based on DNA methylation levels”. By construction, EEAA tracks both age related changes in blood cell composition and intrinsic epigenetic changes. By definition, none of our three measures of epigenetic age acceleration are correlated with the chronological age.

### Estimating blood cell counts based on DNA methylation levels

We estimate blood cell proportions using two different software tools. Houseman's estimation method [45], which is based on DNA methylation signatures from purified leukocyte samples, was used to estimate the proportions of CD8+ T cells, CD4+ T, natural killer, B cells, and granulocytes. Granulocytes are also known as polymorphonuclear leukocytes. The advanced analysis option of the epigenetic clock software [14, 21] was used to estimate the percentage of exhausted CD8+ T cells (defined as CD28-CD45RA-) and the number (count) of naïve CD8+ T cells (defined as (CD45RA+CCR7+).
